# DA-JC1 improves learning and memory by antagonizing Aβ31–35-induced circadian rhythm disorder

**DOI:** 10.1186/s13041-019-0432-9

**Published:** 2019-02-11

**Authors:** Li Wang, Rui Zhang, Xiaohong Hou, Changtu Wang, Shuai Guo, Na Ning, Cong Sun, Yuan Yuan, Lin Li, Christian Hölscher, Xiaohui Wang

**Affiliations:** 1 Department of Pathology, Shanxi Medical University, Taiyuan, People’s Republic of China; 2 Laboratory of Chronobiology, Shanxi Medical University, Taiyuan, People’s Republic of China; 3 Laboratory of Morphology, Department of Basic Medical Sciences, Shanxi Medical University, Taiyuan, People’s Republic of China; 4 Key Laboratory of Cellular Physiology, Shanxi Medical University, Taiyuan, People’s Republic of China; 5 Second Hospital, Shanxi Medical University, Taiyuan, People’s Republic of China; 6 Biomedical and Life Science, Faculty of Health and Medicine, Lancaster University, Lancaster, LA1 4YQ UK

**Keywords:** DA-JC1, Learning and memory, Aβ31–35, Circadian rhythm

## Abstract

Studies have shown that a normal circadian rhythm is crucial to learning and memory. Circadian rhythm disturbances that occur at early stages of Alzheimer’s disease (AD) aggravate the progression of the disease and further reduce learning and memory in AD patients. The novel, dual GLP-1R/GIPR agonist DA-JC1 has been found to exert a stronger hypoglycemic effect than a GLP-1R agonist alone and has been shown to exert neuroprotective effects. However, it is not clear whether DA-JC1 improves the Aβ31–35-induced decline in learning and memory ability by restoring disrupted circadian rhythms. In the present study, we carried out a mouse wheel-running experiment and Morris water maze test (MWM) and found that DA-JC1 could effectively improve the decline of learning and memory and circadian rhythm disorders induced by Aβ31–35. After downregulating Per2 expression via lentivirus-shPer2 in the hippocampus and the hippocampal HT22 cells, we found that circadian rhythm disorders occurred, and that DA-JC1 could not improve the impaired learning and memory. These results suggest that DA-JC1 improves damage to learning and memory by antagonizing circadian rhythm disorders induced by Aβ31–35. The outcome of this ongoing study may provide a novel therapeutic intervention for AD in the future.

**Electronic supplementary material:**

The online version of this article (10.1186/s13041-019-0432-9) contains supplementary material, which is available to authorized users.

## Introduction

According to the World Alzheimer Report 2018, around 50 million people have dementia, and the number is projected to reach 152 million by 2050, of which 50–75% are Alzheimer’s disease (AD) patients. Learning and memory dysfunction in AD patients seriously affect their quality of life [1]. Studies have shown that, as the main pathological change of AD, the deposition of amyloid-β protein (Aβ) in the brain damages learning ability and memory capacity [2]. However, the underlying mechanism is not yet understood.

Studies have shown that normal circadian rhythm is crucial to learning and memory [3]. Circadian rhythm disturbances that occur at early stages of AD aggravate the progression of the disease and further reduce learning and memory ability in AD patients [4, 5]. Circadian rhythm disorders in AD patients manifest as excessive daytime sleepiness and fragmented sleep [6]. The circadian rhythms are daily oscillations in various biological processes that are regulated by a transcription-translation feedback loop (TTFL) composed of circadian clock genes and proteins [7]. Studies have shown that abnormal deposition of Aβ is an important factor for the AD-related disruption of the circadian rhythm [8]. The 5xFAD mouse with high Aβ deposition was found to exhibit abnormal expression of the clock gene Per2 [9]. Our previous results also showed that hippocampal injection of Aβ31–35 caused circadian rhythm disorder in mice and abnormal expression of Per1 and Per2 [10]. So, can we improve the Aβ31–35-induced decline of learning ability and memory capacity by restoring disrupted circadian rhythms?

Studies have shown that AD has many similar pathophysiological characteristics with type 2 diabetes mellitus (T2DM). The insulin signaling pathways are impaired in both T2DM and AD patients [11]. Patients with T2DM have greater impairments in cognitive function [12], and patients with AD are at high risk of developing T2DM [13]. Hence, the T2DM drugs glucagon-like peptide 1 (GLP-1) and glucose-dependent insulin-promoting peptide (GIP) may be used to treat AD. It has been found that the novel, dual GLP-1R/GIPR agonist DA-JC1 has a stronger hypoglycemic effect [14] than GLP-1R agonist Liraglutide. DA-JC1 was found to play a neuroprotective role in Parkinson’s disease mice by reducing synapse loss and motor dysfunction [15], with a longer half-life than a single-receptor agonist [16]. However, it is not clear whether DA-JC1 improves the circadian rhythm disorder and further restores the learning and memory impairment induced by Aβ31–35.

This study aimed to investigate whether DA-JC1 could effectively alleviate the circadian rhythm disorder and impairment of learning and memory induced by Aβ31–35 using the running-wheel and Morris water maze tests. Furthermore, the expression of Per2 was downregulated with lentivirus to explore whether the circadian rhythm was involved in DA-JC1’s improvement of the decline in learning ability and memory capacity induced by Aβ31–35.

## Methods

### Experimental animals

Six-to-eight-week-old male C57BL/6 mice (18–22 g) were provided by the Experimental Animal Center of Shanxi Medical University. The mice were kept at room temperature, relative humidity levels of 35–55%, and free diet. The use of animals in experiments was in accordance with the national experimental animal use regulations.

### Intrahippocampal injection

C57BL/6 mice were anaesthetized with 5% chloral hydrate by intraperitoneal injection. Animal heads were immobilized in a standard stereotaxic instrument according to the anatomical map of the mice, and reagents were injected into the CA1 area of the hippocampus (2.0 mm posterior to bregma, 1.8 mm lateral to midline, and 1.8 mm below the horizontal skull surface) at a uniform speed (0.2 μl/min).

In the Aβ31–35 group, Aβ31–35 was dissolved in tri-distilled water and diluted to 1 μg/μl, and then incubated for 36 h at a constant temperature of 37 °C (water bath). A 7.5 nmol dose of Aβ31–35 was injected into the hippocampus of each mouse [17]. For the control group, the same volume of normal saline was injected. The DA-JC1 pre-administration group received intraperitoneal injections of 50 nmol/kg DA-JC1 for 1 week before intrahippocampal injection of Aβ31–35. The mice in the DA-JC1 group were only administered with an equivalent dose of DA-JC1 intraperitoneally for 1 week [18].

### Morris water maze test

One week after hippocampal injection, the Morris water maze (MWM) test was used to study spatial learning and memory [19]. The water maze is a circular pool with a diameter of 120 cm and a depth of 30 cm. The inside of the water maze is painted black, and the water temperature is 23 ± 2 °C. The pool was divided into four quadrants—northeast (NE), southeast (SE), southwest (SW), and northwest (NW)—and a round escape platform 6 cm in diameter and 24 cm in height was placed in the NW quadrant 1 cm below the water surface. A camera was fixed above the pool and captured the process of mouse swimming. The data were analyzed using Smart 3.0 image software. The MWM test consisted of two types: a place navigation trial and a spatial probe trial. In the place navigation trial, the mouse was placed into the water facing the maze’s wall from a random quadrant; we monitored the animal until it reached the hidden escape platform and recorded the time this took as escape latency. If the animal failed to find the platform in the 1-min period, we placed it back on the platform and allowed it to remain on the platform for 10 s, then removed the animal and put it back in the cage. Mice were trained four times a day at 20-min intervals for five consecutive days. We then conducted probe trials similar to the hidden platform training, with the exception that no platform was in the maze on the sixth day. During testing, the mouse was released from a fixed point and had 60 s to swim in the maze. We tracked the percentage of time the animal spent in each quadrant of the maze.

### HT22 cell culture

An HT22 mouse hippocampal neuronal cell line was purchased from GuangZhou Jennio Biotech Co., Ltd. The HT22 cells were cultured in DMEM complete medium with 10% fetal bovine serum and placed in a stable-temperature incubator at 37 °C, 5% CO_2_, and saturated humidity. The cell synchronization was performed when the cells were completely attached. Complete medium was changed to the starvation medium with 1% fetal bovine serum. After 1 h culture, the circadian rhythm of HT22 cells was regarded synchronized as Circadian Time 0 (CT0). Then the starvation medium was changed to the complete medium, and the synchronized cells were cultured for n hours as CTn [20]. The cells of the control group were cultured in complete medium; the cells of the Aβ31–35 group were cultured in complete medium with 5 μM Aβ31–35; in DA-JC1 + Aβ group, the cells were pretreated with 300 nM DA-JC1 for 1 h, and then 5 μM Aβ31–35 was added; the cells of the DA-JC1 group were cultured in complete medium with 300 nM DA-JC1. Subsequently, the cells were collected at CT4, CT8, CT12, CT16, CT20, and CT24 to detect the expression of the circadian clock genes Per1 and Per2 and learning and memory-associated proteins synaptophysin (SYP) and growth-associated protein 43 (GAP-43).

### Western blot

We collected the cells by centrifugation and performed the lysis with RIPA lysate on ice, and the supernatant liquid was obtained after being centrifuged. We quantified the protein content using a BCA Protein Assay Kit and calculated the sample protein concentrations; then the protein was denatured for 10 min at 100 °C. Samples were added to SDS-PAGE, and proteins were then transferred to PVDF membranes after electrophoresis. The membrane was blocked for 2 h at room temperature with 5% skimmed milk, then incubated with anti-SYP primary antibody (Santa Cruz, sc-17,750), anti-GAP43 primary antibody (Santa Cruz, sc-17,790), anti-PER1 primary antibody (Abcam, ab3443), and anti-PER2 primary antibody(Santa Cruz, sc-25,363) overnight at 4 °C. Subsequently, the membrane was incubated with the corresponding secondary antibody for 2 h at room temperature after TBST washing. The Super ECL Plus ultrasensitive luminescent solution was added, and the image was captured using a gel imaging system. ImageJ was used to analyze the gray value and calculate the relative expression of the corresponding protein.

### Wheel-running behavioral test

C57BL/6 mice were randomly divided into four groups: control group, Aβ31–35 group, DA-JC1 + Aβ group, and DA-JC1 group. The mice of each group were placed in wheel-running device at 22 ± 2 °C and a relative humidity of 35–55%. The animals were exposed to light/dark cycles of 12:12 h (LD) for 1 week, then transferred to constant darkness (DD) for 2 weeks. The endogenous circadian rhythm of mice was represented by circadian time (CT) in the DD condition. The circadian day was divided into 24 equal parts, with each circadian hour representing one CT and the onset of activity defined as CT12 [10]. Running wheel activity was recorded using the VitalView program and analyzed using ActiView software. The free running period and daily activity were the main indicators. After the end of the wheel running, the mice were decapitated at CT4, CT8, CT12, CT16, CT20, and CT24, and then the hippocampus was removed quickly and placed on ice in a dark environment to further detect the expression of Per1 and Per2.

### Real-time PCR

The Per1 and Per2 mRNA expression levels were detected by real-time PCR at different CT time points. Specifically, the total RNA of HT22 cells was extracted using TRIzol and inverted to cDNA. The SYBR Green kit was used, and the corresponding primers were added to specific amplification. Primers were designed as follows: Per1 (Gen-Bank ID NM_001159367.1) forward: 5′-CAGCCGTGCTGCCTACTCATT-3′, reverse: 5′-AGAGGCAGCTTGGTGTGTGTC-3′; Per2 (Gen-Bank ID NM_011066.3) forward: 5′-TGGTCTGGACTGCACATCTGG-3′, reverse: 5′-AGGTCACTTGACGT-GGAGATGG-3′; GAPDH (Gen-Bank ID NM_008084.2) forward: 5′-AAATGGTGAAGGTCGGTGTGAAC-3′, reverse: 5′-CAACAATCTCCACTTTGCCACTG-3′. All gene expression data from each sample were normalized with the expression of GAPDH at CT4 in the control group, and relative mRNA quantification was performed using the 2^-ΔΔCt^ method.

### Detection of Per2 expression and efficiency in mice hippocampus after lentivirus interference

Hanbio Biotechnology Co., Ltd. provided the lentivirus vector construction (LV-shPer2-GFP-PURO, hereinafter abbreviated as LV-shPer2) with GFP- and puromycin-resistant genes. Intrahippocampal injection of LV-shPer2 was used to interfere with Per2 expression in the mouse hippocampus. Mice were anesthetized and fixed in a standard stereotaxic instrument. The LV-shPer2 (1 μl virus per side, 0.2 μl/min) or the LV-NC (nonspecific shRNA lentivirus) was injected slowly into the bilateral hippocampus in the corresponding experimental group [21]. Hippocampal tissues were cut into 30 μm sections, and the slides were observed to confirm the efficiency of infection under a laser scanning confocal microscope. Then, the interference efficiency of Per2 was determined using Western blot. In addition, the mice of each group were subjected to a run-wheel behavioral test and Morris water maze test to detect circadian activity rhythms and learning and memory capacity, respectively.

### Per2 gene silencing and efficiency detection in HT22 cells

Lentivirus LV-shPer2 was used to infect mouse hippocampal HT22 neurons to silence Per2 gene expression. In order to choose a more appropriate amount of virus, we first determined the optimal multiplicity of infection (MOI, the number of virions per cell in a transduction). The optimal MOI corresponds to the number of efficient lentiviral vectors needed per cell in order to obtain the maximal percentage of transduced cells. Suitable lentivirus and auxiliary agent polybrene were added to cells according to MOI. Virus-containing medium was replaced with fresh complete medium at 12–16 h after virus infection. Then the cells were screened after 72 h infection. Uninfected and virus-infected cells with uniform cell density were prepared for the pre-screening, and cells were cultured in complete medium containing puromycin. The culture medium was replaced every other day until all uninfected cells died. Then the virus-infected cells were cultured for 1–2 days in medium containing a 50% concentration of puromycin, the percentage of GFP-positive cells was observed under a laser scanning confocal microscope, and the interference efficiency was assessed by immunofluorescence and Western blot. The expression of Per1, and Per2 and the expression levels of learning and memory-related proteins SYP and GAP-43 were investigated in the LV-shPer2 group as well as the LV-NC group.

### Statistical analysis

Statistical analysis was performed with SPSS 16.0 statistical software. The normal distribution of measured data was expressed as mean ± SEM. ActiView Biological Rhythm Analysis was used to analyze data from the wheel-running assay. JTK_CYCLE was used to detect the rhythmic expression of clock genes [22]. One-way analysis of variance (ANOVA) was used for multiple group comparisons, and a least significant difference (LSD) *t* test was used for comparison between groups. The significance level of α = 0.05, *P* < 0.05 was considered statistically significant.

## Results

### DA-JC1 improved the impairment of learning and memory capacity in C57BL/6 mice induced by Aβ31–35

**Fig. 1:**
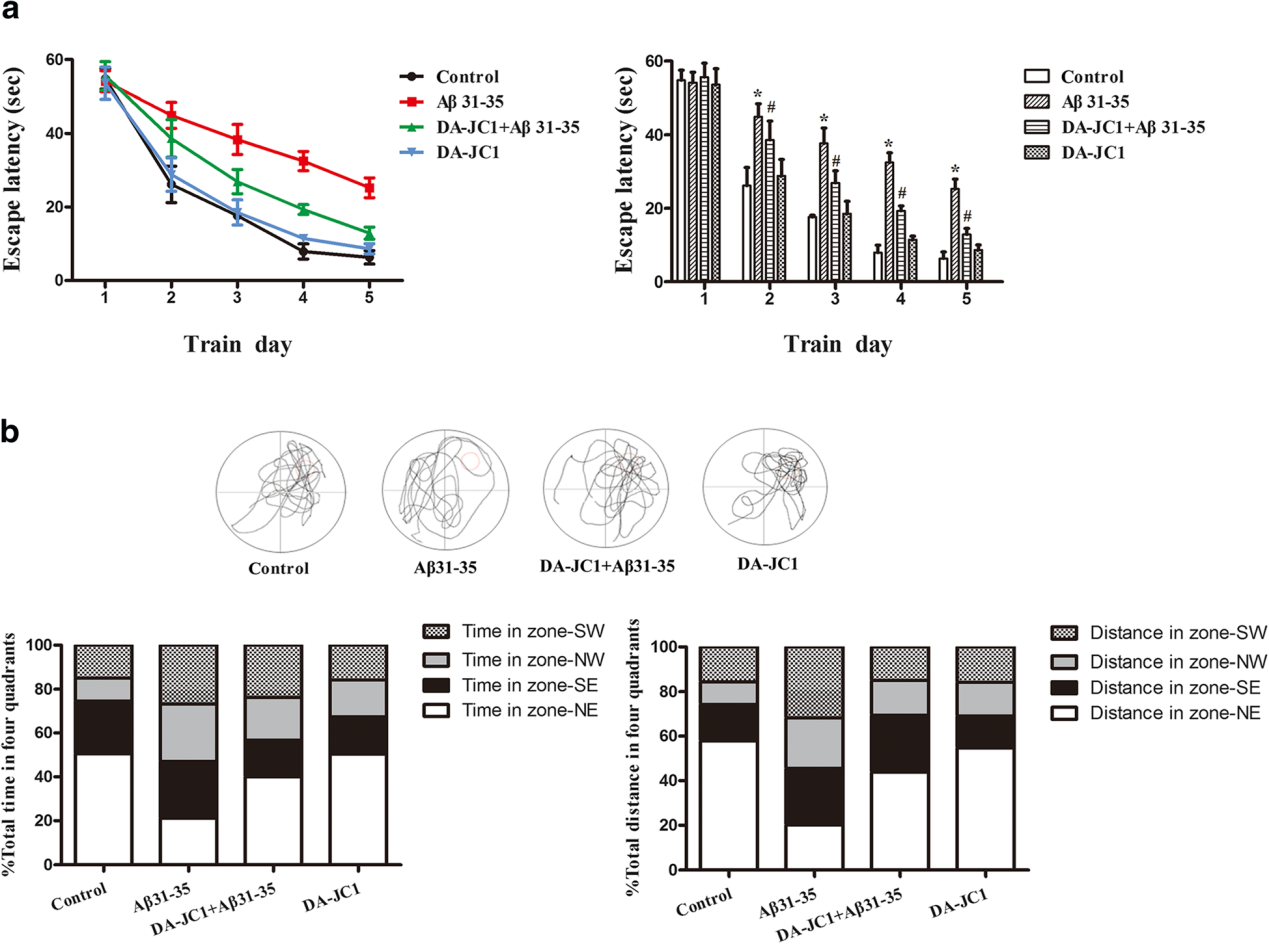
The effect of DA-JC1 on Aβ31–35-induced abnormal learning and memory capacity in mice shown by the Morris water maze test. (**a**) Hidden platform acquisition tests (4 trials per animal per day for 5 consecutive days) were performed, and the escape latency at each day is shown for each group of mice. (**b**) Representative swimming trajectories of mice on the sixth training day after finishing the hidden platform test and the percentage of time spent and distance traveled in the four quadrants. Zone-NE indicates the target quadrant. Data are expressed as means ± SEM (*n* = 8 per group). *P < 0.05 compared with the control group; #*P* < 0.05 compared with the Aβ31–35 group

To explore the effect of hippocampal injection of Aβ31–35 on the learning and memory capacities of mice, we used the Morris water maze test to observe the mouse swimming path, escape latency, percentage of time spent in the target quadrant, and percentage of distance traveled in the target quadrant. As shown in Fig. 1a, the escape latency gradually decreased in all groups after 5 days of acquisition training in the water maze. Compared with the control group, the escape latency of mice injected with Aβ31–35 in the hippocampus was significantly prolonged, indicating that Aβ31–35 could induce the decline of spatial learning ability in the hidden platform test. We also found that intrahippocampal injection of Aβ31–35 decreased the percentage of time and swim distance spent in the target quadrant, demonstrating that Aβ31–35 could induce spatial memory deficits (Fig. 1b). In addition, the escape latency was significantly shorter than that of the Aβ31–35 group after pretreatment with intraperitoneal injection of DA-JC1 (Fig. 1a). The percentage of distance traveled in the target quadrant and the time spent in the target quadrant were significantly prolonged (Fig. 1b), suggesting that DA-JC1 could effectively prevent Aβ31–35-induced attenuation of spatial learning and memory capacity in C57BL/6 mice.

### DA-JC1 reversed the Aβ31–35-induced decline in the expression of SYP and GAP-43 in HT22 hippocampal neuronal cells

**Fig. 2:**
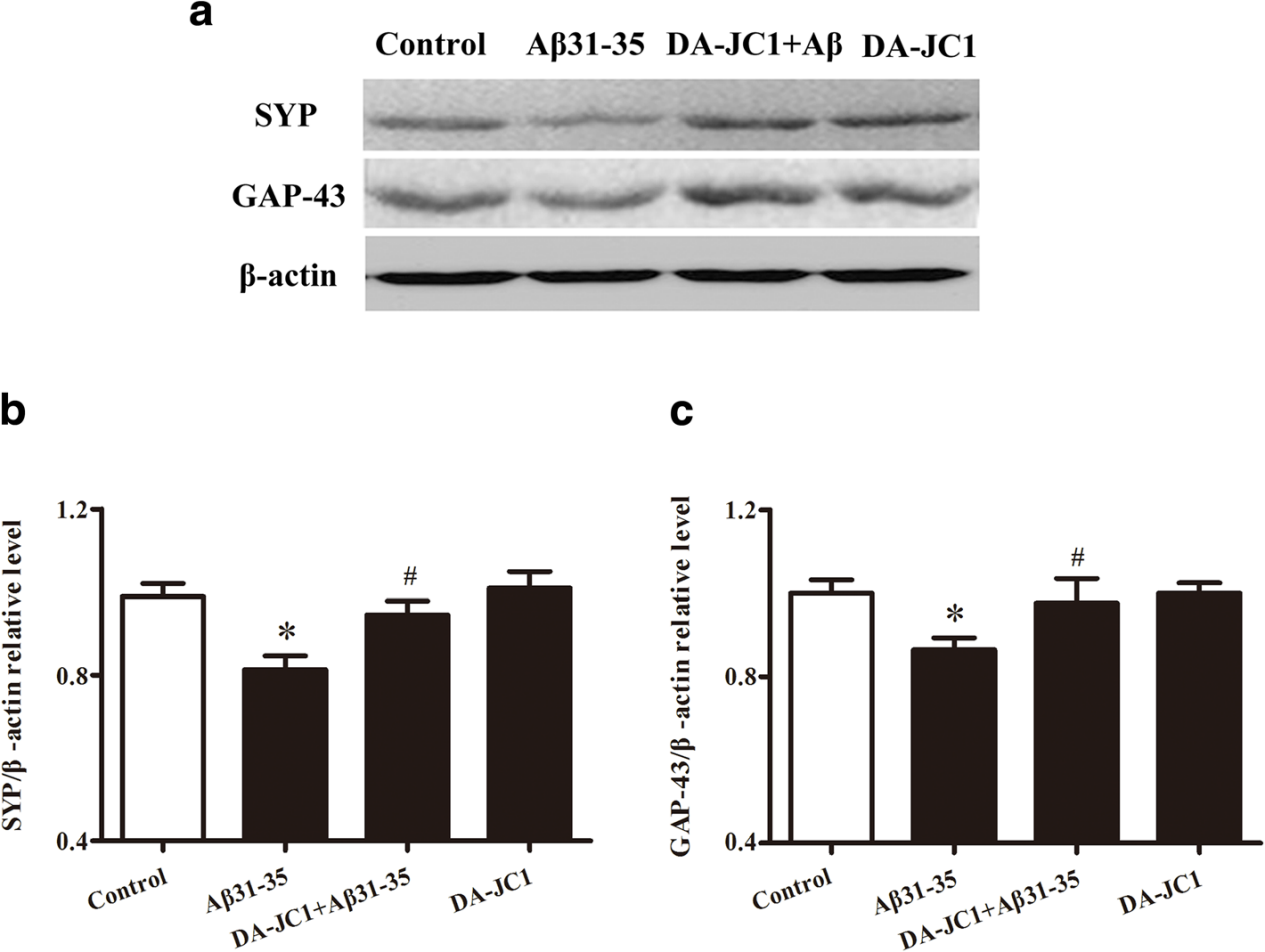
The influence of DA-JC1 on Aβ31–35-induced decreased expression of SYP and GAP-43 in HT22 hippocampal neuronal cells. (**a**) Representative immunoblots show the levels of SYP and GAP-43 in HT22 cells. (**b**) Quantification analysis of SYP from a. (**c**) Quantification analysis showing the protein expression of GAP-43. Data are expressed as means ± SEM (*n* = 6 per group). *P < 0.05 compared with the control group; #*P* < 0.05 compared with the Aβ31–35 group

SYP, a major synaptic vesicle protein, is an indicator of changes in synaptic plasticity [23]. GAP-43, a neural-specific protein, plays a significant role in synaptic remodeling, which forms the basis of learning and memory [24]. In the present study, Western blot was used to detect the protein expression of SYP and GAP-43 in HT22 cells, and the data showed that Aβ31–35 could decrease SYP and GAP-43 protein expression compared with the control group. Pretreatment with DA-JC1 significantly reversed the decline in the expression of SYP and GAP-43, and there was no significant change in the DA-JC1 group (Fig. 2). These results showed that DA-JC1 upregulated the decreased expression of learning- and memory-related proteins such as SYP and GAP-43 induced by Aβ31–35 in HT22 cells.

### DA-JC1 antagonized the Aβ31–35-induced a change in the circadian rhythm of C57BL/6 mice

**Fig. 3:**
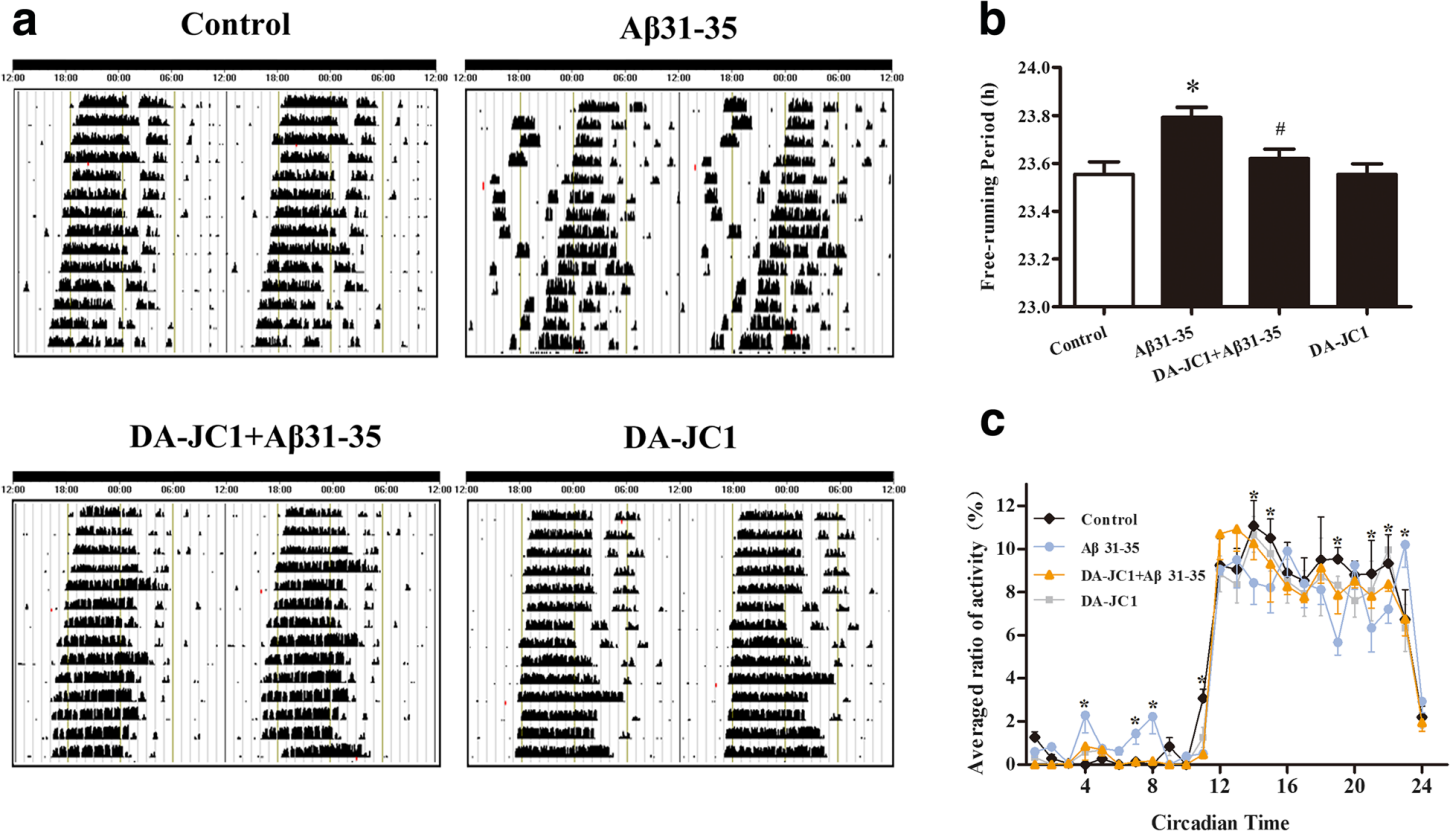
The effect of DA-JC1 on Aβ31–35-induced circadian rhythm disorder in mice. (**a**) Representative locomotor activity records of each group. (**b**) The free-running period of the locomotor activity rhythm in each group. (**c**) The average activity in DD of all the mice at each one-hour interval in the actogram. Data were expressed as means ± SEM (n = 6 per group). *P < 0.05 compared with the control group; #*P* < 0.05 compared with the Aβ31–35 group

Wheel-running experiments were performed to explore the effect of Aβ31–35 on the endogenous circadian rhythm of C57BL/6 mice, and the results showed that mice in the control group displayed rhythmic running wheel activity under a DD environment. The movement phase and the resting phase were clearly demarcated, and the activities mainly occurred during subjective nights. Intrahippocampal injection of Aβ31–35 induced a change in the circadian rhythm, as shown by an unclear movement phase and resting phase (Fig. 3a, c) and a prolonged free running period compared with the control group (Fig. 3b). After intraperitoneal injection of DA-JC1, the movement/rest phases became more obvious, and activities were mainly concentrated in the subjective night (Fig. 3a, c), suggesting that DA-JC1 pretreatment partially restored the circadian rhythm disorder in mice. Compared with the Aβ31–35 group, the free running period was shortened (Fig. 3b). Taken together, these results showed that DA-JC1 pretreatment could antagonize Aβ31–35-induced circadian rhythm disorder in C57BL/6 mice.

### DA-JC1 partially reversed the abnormal expression of Per1 and Per2 in the hippocampus induced by Aβ31–35

**Fig. 4:**
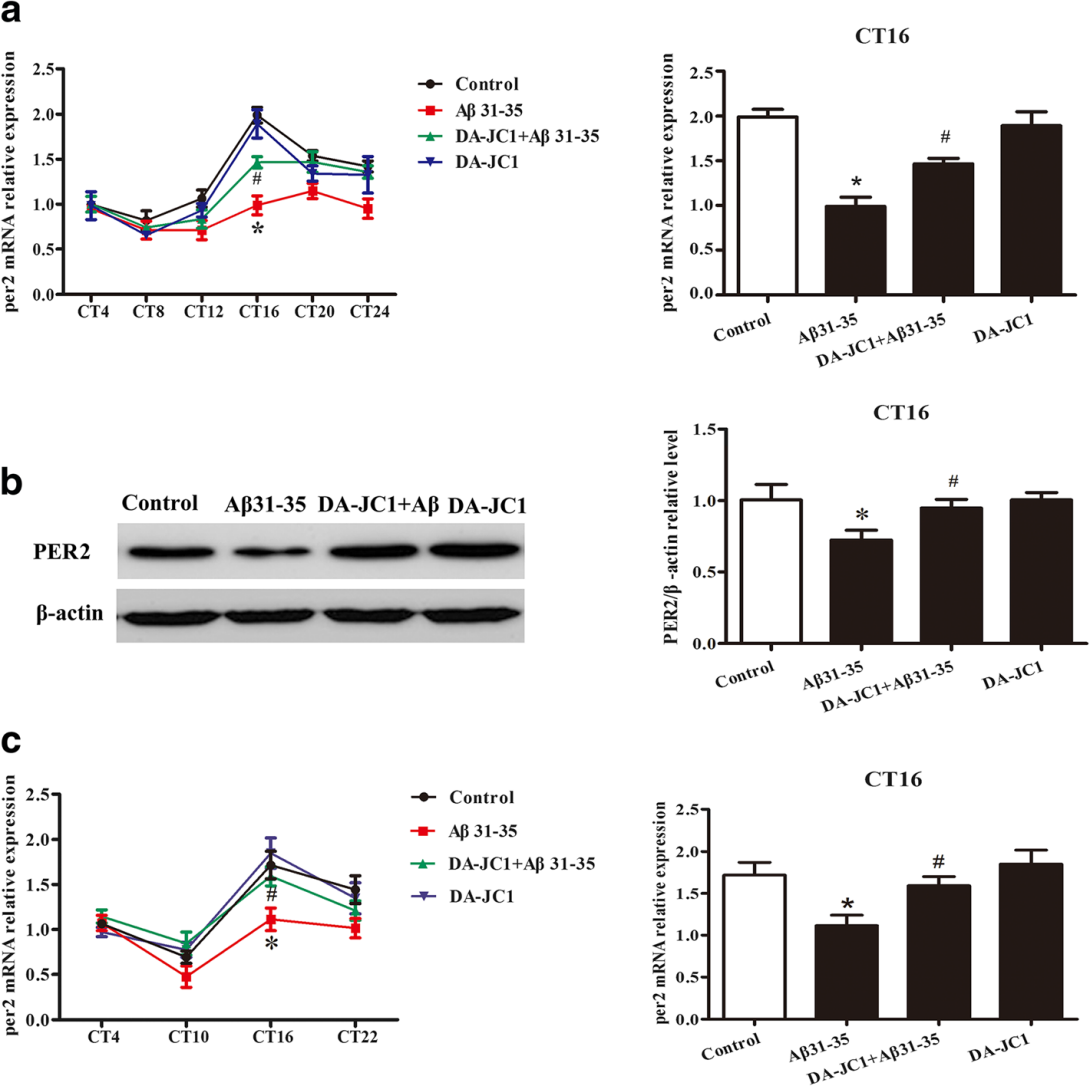
The effect of DA-JC1 on Aβ31–35-induced abnormal expression of Per2 in HT22 hippocampal cells. (**a**) mRNA levels of Per2 were assayed at indicated circadian times. (**b**) Representative immunoblots show the levels of Per2 at CT16. Quantifications of Western blots were obtained by using β-actin as a loading control. (**c**) mRNA expression of Per2 in the hippocampal tissue at different time points was detected. Data are expressed as means ± SEM (n = 6 per group). *P < 0.05 compared with the control group; #*P* < 0.05 compared with the Aβ31–35 group

The expression of Per1 and Per2 genes in HT22 cells was detected by real-time PCR. JTK_CYCLE was used to detect rhythmic mRNA expression of Per1 and Per2. The results showed that Per1 mRNA expression did not have a circadian rhythm (Additional file 1: Figure S1), while Per2 mRNA expression had a circadian rhythm (Fig. 4a). The Per1 mRNA level was significantly decreased at CT12, and the Per2 mRNA level was significantly decreased at CT16 after exposure to 5 μM Aβ31–35. The mRNA levels of Per1 at CT12 and Per2 at CT16 showed an obvious increase after pretreatment with DA-JC1 for 1 h compared with the Aβ31–35 group. There was no significant difference in the levels of Per1 and Per2 mRNA expression between the DA-JC1 alone group and control group (Additional file 1: Figure S1a, Fig. 4a). Then we further examined the expression of PER1 protein at CT12 and Per2 protein at CT16. The data showed that the expression of PER1 and PER2 proteins was significantly lower in the Aβ31–35 group, while pretreatment with DA-JC1 could restore the decline of PER1 and PER2 proteins. Similarly, DA-JC1 alone had no significant effect on the relative expression of PER1 and PER2 proteins (Additional file 1: Figure S1b, Fig. 4b).

Furthermore, in order to prove that the hippocampal clock was involved in the disruption by Aβ31–35 and improvement by DA-JC1, the mRNA expression of Per2 in the hippocampal tissue was detected. The results showed that Per2 mRNA expression exhibited circadian rhythms at different time points in the control group, showing a trough at CT10 and a peak at CT16. The expression of Per2 mRNA was remarkably decreased by Aβ31–35 and recovered after pretreatment with DA-JC1 for 1 week compared with the Aβ31–35 group at CT16 (Fig. 4c). These results suggested that DA-JC1 could significantly ameliorate the abnormal protein expression of Per1 and Per2 induced by Aβ31–35 in the hippocampus.

### Interference with Per2 by lentivirus caused the circadian rhythm disorder in mice

**Fig. 5:**
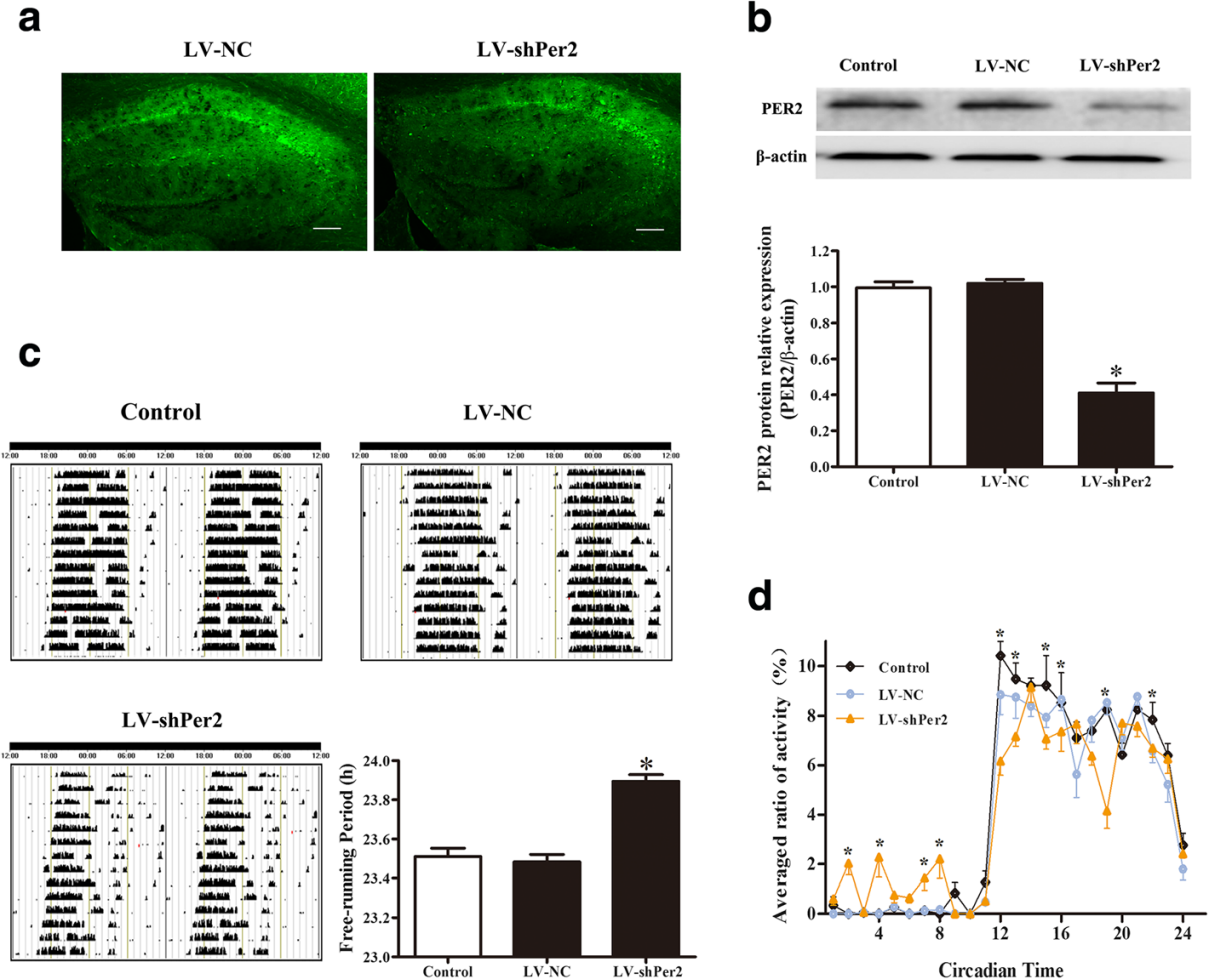
The role of Per2 interference using lentivirus on circadian rhythm disorder in mice. (**a**) A laser scanning confocal microscope was used to observe the GFP-positive cells. Scale bar = 100 μm. (*n* = 3 per group). (**b**) Representative Western blot and quantification analysis showing the protein expression of Per2. (*n* = 5 per group). (**c**) Representative locomotor activity records of each group and the free-running period of the locomotor activity rhythm in each group. (n = 5 per group). (**d**) The point-by-point average activity in DD of all the mice at each one-hour interval in the actogram. (n = 5 per group). Data are expressed as means ± SEM. *P < 0.05 compared with the control group

Lentiviral vectors expressing shRNA were used to suppress Per2 gene expression in mouse hippocampal tissue after intrahippocampal injection, and then voluntary wheel running was used to assess mouse circadian rhythms. Mice were randomly divided into the control group, negative control virus (LV-NC) group, and virus (LV-shPer2) group. Hippocampal tissue sections of each group were taken and observed under a laser confocal microscope. The GFP-positive cells were observed in the LV-shPer2 group (Fig. 5a) and LV-NC group (Fig. 5a). Then Western blot was used to detect the knockdown efficiency of the Per2 gene, and the results showed that, compared with the control group, Per2 expression in the hippocampus of LV-NC mice did not change significantly, whereas LV-shPer2 significantly decreased Per2 protein expression (Fig. 5b), indicating that shPer2 lentivirus transfection effectively reduced Per2 expression.

Furthermore, a voluntary wheel running behavioral test was used to measure circadian activity rhythms in Per2-deficient mice. The results showed that the suppression of mouse Per2 expression in the hippocampus caused a change in the running activity rhythm, which was manifested as movement fragmentation and abnormal moving and resting phases (Fig. 5c). Statistics showed that the free-running period increased (Fig. 5c) and the locomotor activity was irregular compared to the control group (Fig. 5d), indicating that circadian rhythm disorder had occurred in mice after interference in the Per2 gene.

### The Per2 shRNA lentivirus weakened the expression of Per1 in HT22 cells

**Fig. 6:**
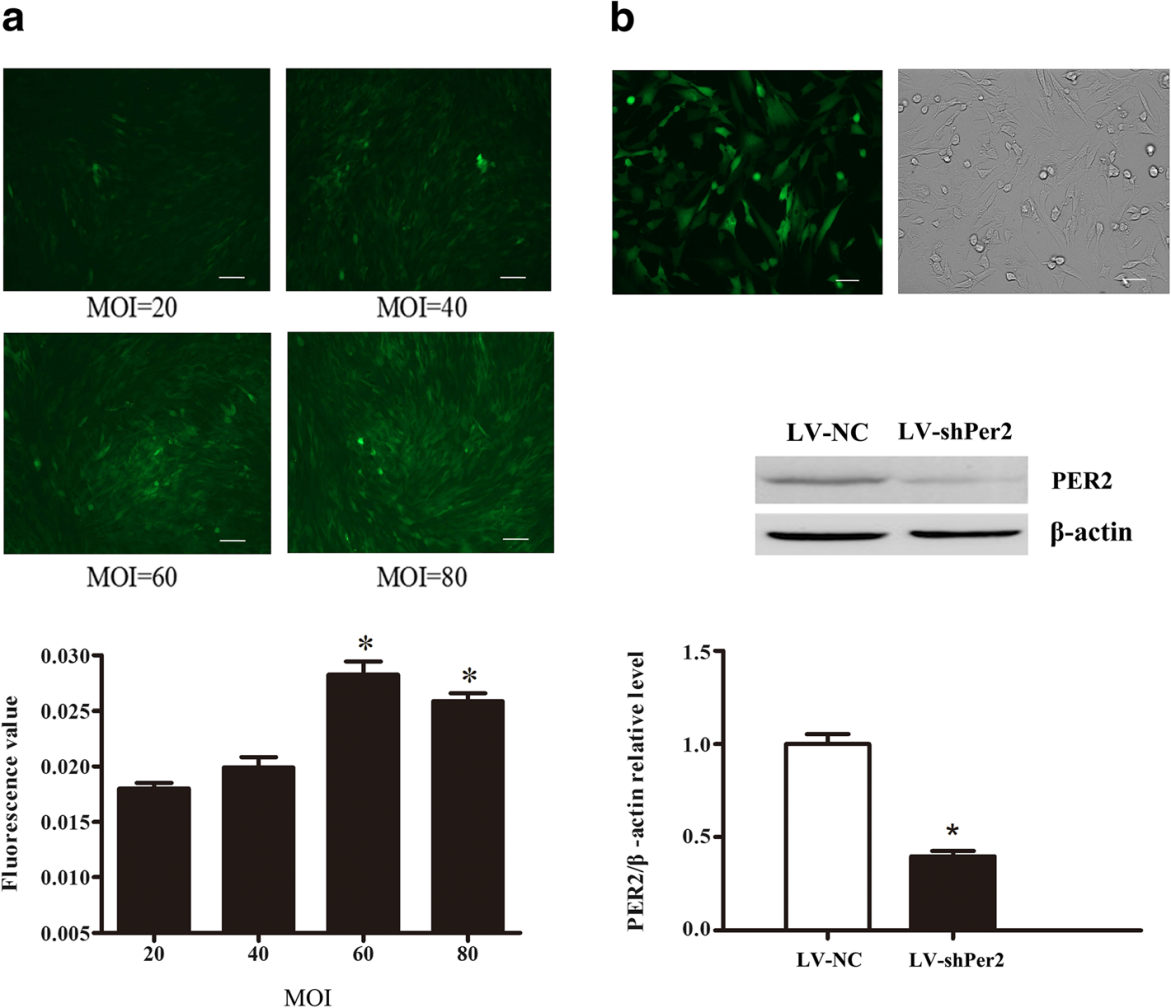
The change in the expression of Per2 after interference with Per2. (**a**) A fluorescence microscope was used to determine the optimal multiplicity of infection (MOI). Scale bar = 100 μm. (**b**) Typical diagram of a fluorescence microscope at an MOI of 60, representative Western blot and quantification analysis of PER2. Scale bar = 50 μm. Data are expressed as means ± SEM (n = 6 per group). *P < 0.05 compared with the control group

HT22 cells were infected with LV-shPer2 to interfere with the expression of Per2. We first determined the optimal multiplicity of infection (MOI), and the results showed that the fluorescence intensity at an MOI of 60 was higher than that at 20 MOI (Fig. 6a). Then we used Western blot to detect Per2 protein at MOI 60. The protein expression of Per2 in HT22 cells infected with LV-shPer2 was reduced remarkably (Fig. 6b), suggesting that LV-shPer2 could suppress the protein expression of Per2 effectively. Further, real-time PCR was used to detect the mRNA expression of Per1, and the expression of Per1 mRNA declined significantly after silencing of Per2 expression in HT22 cells (Data not shown), suggesting that suppression of Per2 expression through RNA interference (RNAi) disrupted the circadian rhythm in HT22 cells.

### Interference with Per2 by lentivirus caused decreased learning and memory abilities in mice

**Fig. 7:**
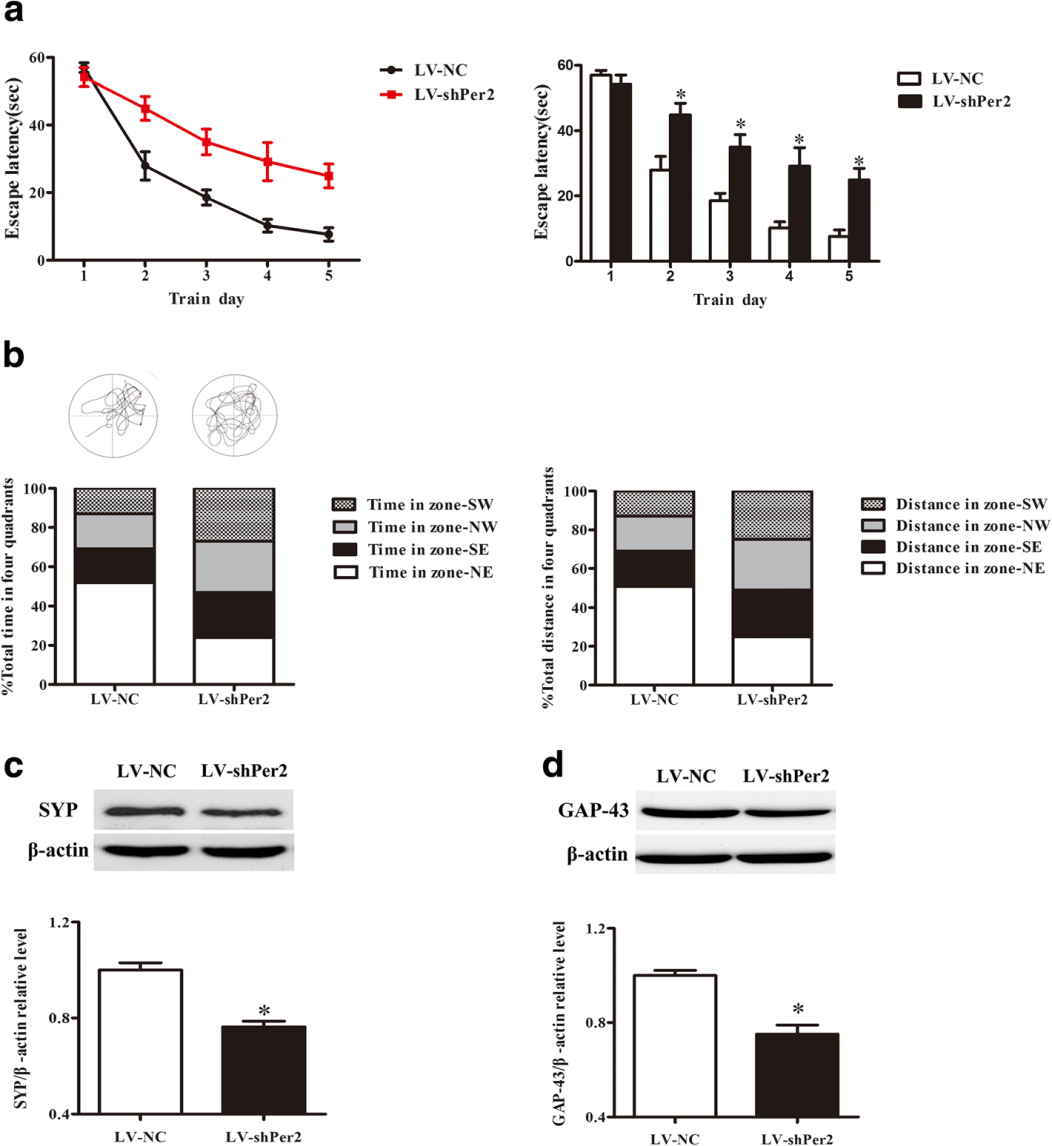
The change in learning and memory abilities after interference in Per2 using lentivirus. (**a**) The escape latency in hidden platform acquisition testing on each day is shown for each group of mice. (**b**) Representative swim paths of mice on the sixth training day after finishing the hidden platform test and the percentage of time spent and distance traveled in the four quadrants. Zone-NE indicates the target quadrant. (**c**) Representative Western blot and quantification analysis of SYP. (**d**) Representative Western blot and quantification analysis of GAP-43. Data are expressed as means ± SEM (n = 6 per group). *P < 0.05 compared with the control group

To investigate the effect of circadian rhythm disorder on learning and memory capacities, a Morris water maze test was performed after Per2 expression was suppressed by lentivirus-mediated RNA interference in mice. The results showed that the escape latency in Per2-deficient mice was significantly longer than that in the control group (Fig. 7a), and the percentage of time spent in the target quadrant and the percentage of distance traveled in the target quadrant decreased significantly compared with the control group (Fig. 7b). Furthermore, the protein expression of SYP and GAP-43, which can reflect learning and memory capacities, were detected in HT22 cells infected with LV-shPer2. The results showed that the expression of SYP and GAP-43 was significantly decreased after suppression of Per2 expression (Fig. 7c). Taken together, these results suggested that silencing of Per2 expression in the hippocampus of mice caused decreased learning and memory capacity and declined expression of learning and memory-related proteins SYP and GAP-43.

### DA-JC1 could not improve the abnormal learning and memory capacity induced by Aβ31–35 after silencing of Per2 expression in the hippocampus of mice

**Fig. 8:**
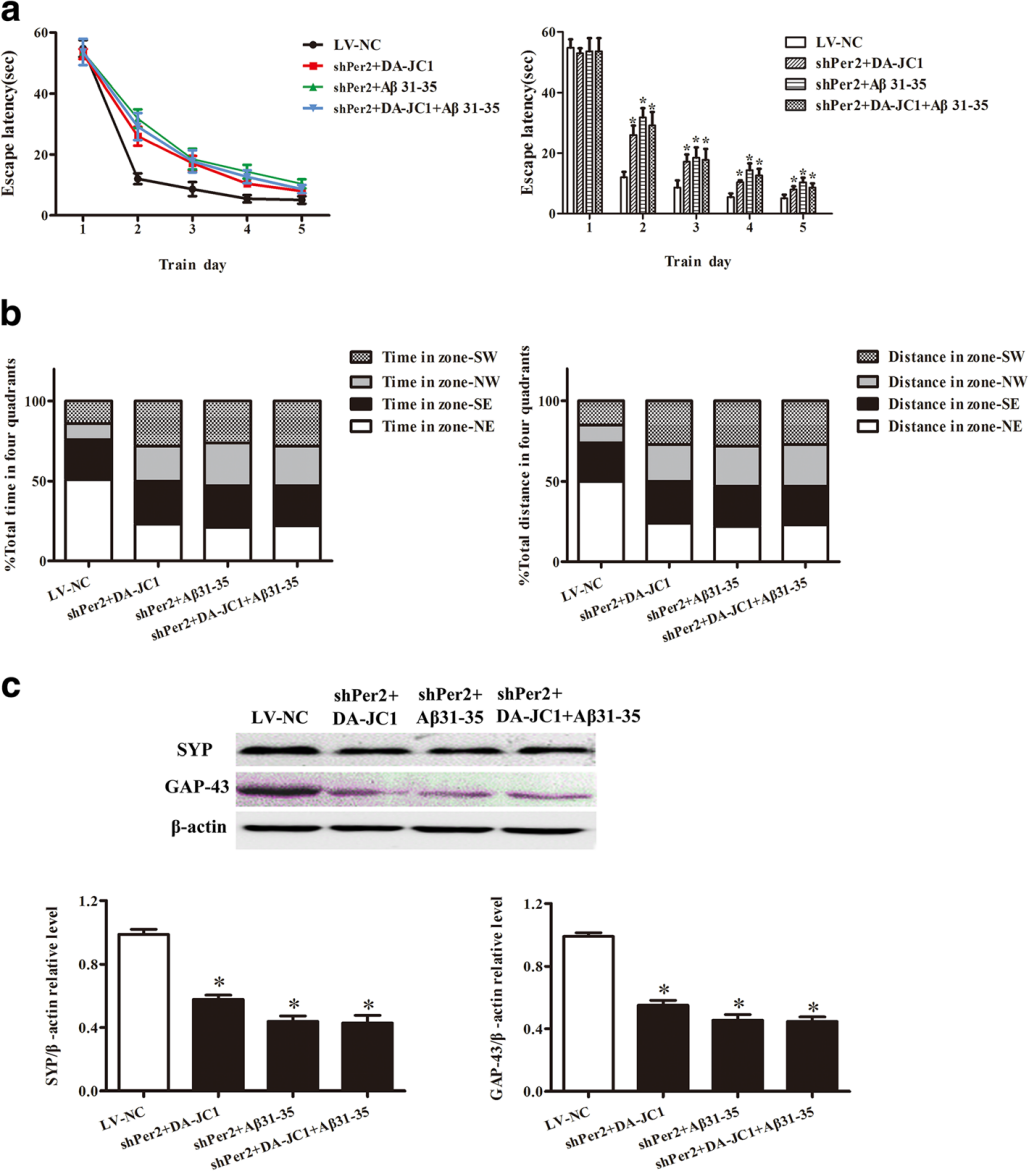
The effect of interference in Per2 using lentivirus on DA-JC1’s improvement of abnormal learning and memory capacity induced by Aβ31–35. (**a**) The escape latency in hidden platform acquisition testing at each day. (**b**) The percentage of time spent and the percentage of distance traveled in the four quadrants. Zone-NE indicates the target quadrant. (**c**) Representative Western blot and quantification analysis of SYP and GAP-43. Data are expressed as means ± SEM (n = 6 per group). *P < 0.05 compared with the control group

To explore the role of circadian rhythm in DA-JC1’s improvement of the Aβ31–35-induced decline in learning and memory capacity of mice, LV-shPer2 was used to suppress the protein expression of Per2 and further disrupt the circadian rhythm. Mice were randomly divided into four groups: the control group, the shPer2 + Aβ31–35 group, the shPer2 + DA-JC1 group, and the shPer2 + DA-JC1 + Aβ31–35 group. The escape latency of the shPer2 + DA-JC1 group and the shPer2 + Aβ31–35 group was prolonged (Fig. 8a), and the percentage of time spent in the target quadrant and the percentage of distance traveled in the target quadrant were reduced significantly compared with the control group (Fig. 8b). However, there was no obvious difference in escape latency, the percentage of time spent in the target quadrant, or the percentage of distance traveled in the target quadrant between the shPer2 + DA-JC1 + Aβ31–35 group and shPer2 + Aβ31–35 group (Fig. 8a, b), indicating that DA-JC1 could not improve the Aβ31–35-induced decline in learning and memory in mice after interference with Per2 by lentivirus.

Similarly, the protein expression levels of SYP and GAP-43 were significantly lower in the shPer2 + DA-JC1 group and shPer2 + Aβ31–35 group than in the control group, and there was no significant difference between the shPer2 + DA-JC1 + Aβ31–35 group and shPer2 + Aβ31–35 group (Fig. 8c), suggesting that Per2 gene silencing resulted in the inability of DA-JC1 to improve the decreased expression of SYP and GAP-43 protein in HT22 cells induced by Aβ31–35.

## Discussion

In the present study, the results of mouse wheel running and the Morris water maze test showed that the new, dual GLP-1 and GIP receptor agonist DA-JC1 could improve the reduced learning and memory capacity of mice and circadian rhythm disorder induced by Aβ31–35. After downregulating Per2 expression via lentivirus-mediated knockdown of the Per2 gene in the hippocampus and the hippocampal HT22 cells, we found that DA-JC1 could not improve the learning and memory impairment induced by Aβ31–35 in mice. Therefore, we believe that DA-JC1 mitigated Aβ31–35-induced learning and memory impairment by improving circadian rhythm disorder.

Studies have shown that AD patients experience progressive learning and memory deficits [1], and the abnormal deposition of Aβ in the brain causes significant learning and memory impairment in mice [25]. Our study also confirmed that, compared with the control group, Aβ31–35 led to prolonged escape latency and decreased time spent and traveled distance in the target quadrant in the Morris water maze test, suggesting that Aβ31–35 impaired learning and memory capacity in C57BL/6 mice.

Extensive evidence has shown that learning and memory impairment in AD is mainly caused by synaptic dysfunction and abnormal hippocampal synaptic plasticity [26]. SYP, a major calcium-binding protein of the synaptic vesicle membrane, accurately reflects the distribution, number, and density of synapses. Y-maze experiments have shown that increased hippocampal SYP levels contribute to new object recognition and learning and memory improvement in mice [27]. GAP-43, a neuron-specific protein, is located in the axon and maintains synaptic morphology [28]. GAP-43 plays an important role in synaptic remodeling, which forms the basis of learning and memory [24]. Studies have shown that the level of SYP and GAP-43 protein expression decreases and synaptic dysfunction occurs in the brains of AD patients [29]. The results of our study also confirmed that SYP and GAP-43 proteins were significantly reduced by Aβ31–35 in HT22 cells, indicating that Aβ could induce the learning and memory dysfunction in AD. However, there is no effective treatment to improve learning and memory.

Numerous studies have shown that a normal circadian rhythm is essential for learning and memory maintenance [3]. C57BL/6 mice kept in a normal light-dark cycle can reach the hidden platform more easily in the Morris water maze [3]. Under constant light conditions, the Morris water maze test showed that mice with circadian rhythm disorders exhibit spatial learning impairments [30]. The biological circadian rhythm system is regulated by a transcriptional feedback loop (TTFL) [31]. The rhythmic expression of circadian genes such as Per1, Cry1, and Bmal1 is weakened in Per2 mutant mice [32]. Research has shown that circadian rhythm disorder often occurs very early in AD patients [33]. Furthermore, studies have found that Tg2576 transgenic mouse models of AD exhibit wheel-running rhythm disruption, a prolonged free-running period [34], and abnormal expression of circadian-clock genes under constant darkness conditions [35]. Studies have shown that Aβ could reduce the transcription of Per2 by degradation of BMAL1 and CBP [9], which is consistent with our findings in the present study. In this study, we found that intrahippocampal injections of Aβ31–35 induced unclear movement/rest phases and a longer free running period than that in control group, as shown by a mouse running wheel behavioral experiment. We also detected the expression of Per1 and Per2 in HT22 cells after Aβ31–35 exposure and found that Aβ31–35 could induce abnormal expression of Per1 and Per2 genes, showing that the expression of Per1/Per2 mRNA and protein decreased significantly at CT12(Per1)/CT16(Per2). Indeed, the expression peaks of the protein and the mRNA should have a time lag. We assumed that the reason why the expression of both Per1 mRNA and PER1 protein differed significantly in CT12 was that Per1 mRNA was measured every four hours. In order to further explain the relationship between the Per1 mRNA and PER1 protein, we added the CT10 time point and found that Aβ31–35 caused a significant decrease in Per1 mRNA expression at CT10. Decreased PER1 protein levels induced by Aβ31–35 were observed at CT12. Hence, there is a time lag between the decreased PER1 protein and Per1 mRNA. Similarly, we added the CT14 time point and found that the expression of Per2 mRNA was remarkably decreased by Aβ31–35 (Additional file 2: Figure S2). Hence, the decrease of Per2 mRNA and PER2 protein has a time lag. Further, JTK_CYCLE found that Per1 mRNA expression in HT22 cells did not have a circadian rhythm and Per2 mRNA expression had a circadian rhythm, so we decided to focus our attention on the role of Per2. Per2 mRNA expression in hippocampal tissue exhibited circadian rhythms in the control group and was remarkably decreased by Aβ31–35. These results demonstrated that Aβ31–35 could disrupt the circadian rhythm in mice.

In order to confirm the role of circadian rhythm disorder in learning and memory, LV-shPer2 was used to suppress the expression of the Per2 gene. A previous study showed that Per2-mutant mice exhibited abnormal long-term potentiation (LTP) in the hippocampus [36]. In the present study, the results of a wheel-running test in a DD environment showed that downregulation of circadian clock gene Per2 in the hippocampus caused significant circadian rhythm abnormalities. After silencing Per2 with LV-shPer2 in HT22 cells, we found that the expression of Per1 mRNA was reduced remarkably. These results suggested that silencing of Per2 expression in the hippocampus and HT22 cells led to disturbance of the endogenous circadian rhythm and abnormal expression of clock genes. The Morris water maze test and SYP/GAP-43 protein detection showed that the change in the circadian rhythm induced by Per2 deficiency resulted in the decline of learning and memory capacity in mice. A previous study showed that Per1 is essential for the formation of memory [21]. According to the paper, deletion of HDAC3 in the hippocampus, which increases acetylation at the Per1 promoter and expression of Per1 mRNA in response to learning, does not affect the circadian rhythm of young or old mice, indicating that the Per1 gene in the hippocampus could regulate synaptic plasticity and memory formation in a circadian rhythm-independent manner [21]. Whether Per2 in the hippocampus affects learning and memory in a circadian rhythm-independent way will be another interesting topic worth researching further. In addition, it has been reported that hippocampal Per1 plays a critical in memory formation by modulating CREB phosphorylation [37]. The damaged memory capacity in Per2-mutant mice is due to the downregulation of p-CREB [36]. Several studies have shown that Aβ could modulate the p-CREB expression in the hippocampus [38]. Therefore, we speculated that CREB phosphorylation was involved in the regulation of Per1 and Per2 expression on learning and memory in hippocampus.

At present, there is no effective treatment for the circadian rhythm disorder in AD patients. The potential relationship between AD and T2DM has been confirmed in recent years [39], and researchers have tried to apply insulin-based treatment strategies for T2DM, such as GLP-1 and GIP analogs, to AD [11]. GLP-1 and GIP exert their effects by binding to their specific receptors, the GLP-1 receptor (GLP-1R) and GIP receptor (GIPR), which are widely distributed in the brain [40]. Our previous experiments also demonstrated that Exendin-4 could improve the Aβ31–35-induced deterioration of learning and memory function [17]. However, the GIP analogue D-Ala2-GIP could reduce the Aβ plaque burden in the brain [41] and improve the learning and memory dysfunction of APP/PS1 double transgenic mice [42]. DA-JC1 is a novel dual GLP-1R/GIPR receptor agonist that simultaneously activates GLP-1R and GIPR and has equal affinity for both receptors [43]. Compared with the GLP-1R agonist, DA-JC1 has a stronger effect on promoting insulin release and lowering blood glucose [14]. Studies have demonstrated that DA-JC1 exerts a neuroprotection on MPTP-induced Parkinson’s disease model mice [16]. However, whether DA-JC1 can alleviate the Aβ-induced decline in learning and memory abilities and circadian rhythm disorder has not been reported. A previous study showed that, as a GLP-1 receptor agonist, liraglutide could attenuate the overexpression of PER1, PER2, and CRY1 protein and partially restore the circadian of T2DM mice [44]. Our previous study showed that Exendin-4 improved Aβ31–35-induced circadian rhythm disorder and learning and memory dysfunction in mice [17]. The present study demonstrated for the first time that DA-JC1 could improve the learning and memory impairment and circadian rhythm disorder in mice induced by Aβ31–35. Specifically, DA-JC1 shortened the escape latency in the Morris water maze and prolonged the percentage of time spent and distance traveled in the target quadrant. Meanwhile, DA-JC1 significantly increased the expression of learning- and memory-related proteins SYP and GAP-43 in HT22 cells. In addition, the results of the running wheel test showed that pretreatment with DA-JC1 improved the circadian rhythm disorder of mice and restored the abnormal expression of Per1 and Per2 clock genes in HT22 cells and Per2 mRNA in mouse hippocampal tissue. Further, we used lentiviral vectors expressing shRNA to downregulate Per2 expression and found that DA-JC1 could no longer improve the learning and memory impairment of mice induced by Aβ31–35 after silencing of Per2 expression, indicating that Per2 in the hippocampus was involved in DA-JC1’s improvement of Aβ31–35-induced learning and memory impairment in mice. The underlying mechanisms behind DA-JC1 cannot improve learning and memory defects without Per2 require further study. The outcome of this ongoing research may provide a novel therapeutic intervention for AD in the future.

## Additional files

Additional file 1: **Figure S1.** (TIF 1891 kb)

Additional file 2: **Figure S2.** (TIF 1272 kb)

## Abbreviations

AD: Alzheimer’s disease
Aβ: amyloid-β protein
CT: Circadian time
DD: Constant darkness
GAP-43: Growth-associated protein 43;
GIP: Gastroinhibitory intestinal peptide
GIPR: GIP receptor
GLP-1: Glucagon-like peptide-1
GLP-1R: GLP-1 receptor
LD: Light-dark
MOI: Multiplicity of infection
MWM: Morris water maze test
RNAi: RNA interference
SYP: Synaptophysin
T2DM: Type 2 diabetes mellitus
TTFL: Transcription-translation feedback loop
