# Supplementary figures and images for: DA-JC1 improves learning and memory by antagonizing Aβ31–35-induced circadian rhythm disorder
Source: Mol Brain. 2019 Feb 11;12:14. doi: 10.1186/s13041-019-0432-9 (PMC6371467; doi:10.1186/s13041-019-0432-9)

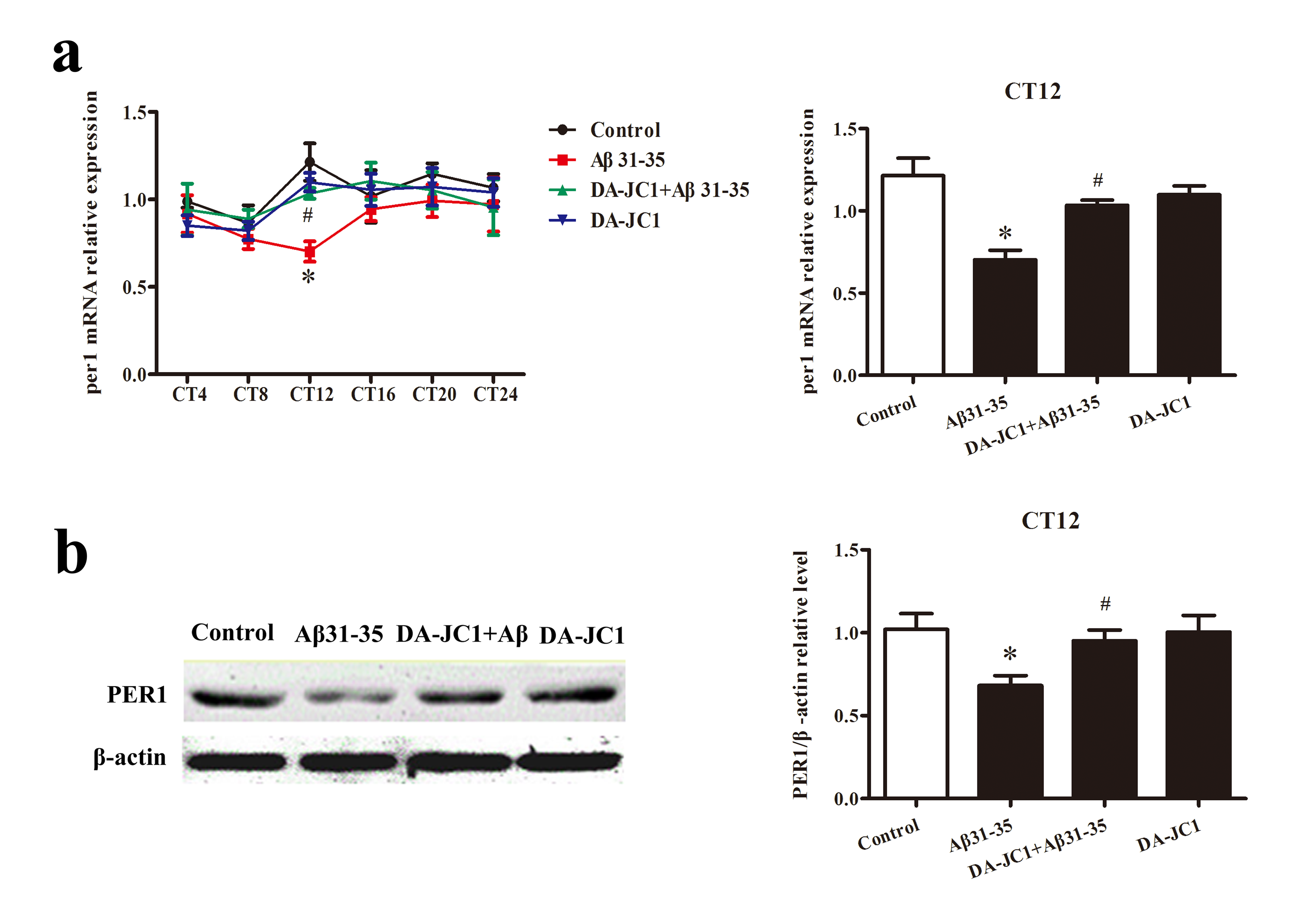

Supplement: Supplementary file 1 — Figure S1. (TIF 1891 kb) [file 13041_2019_432_MOESM1_ESM.tif]

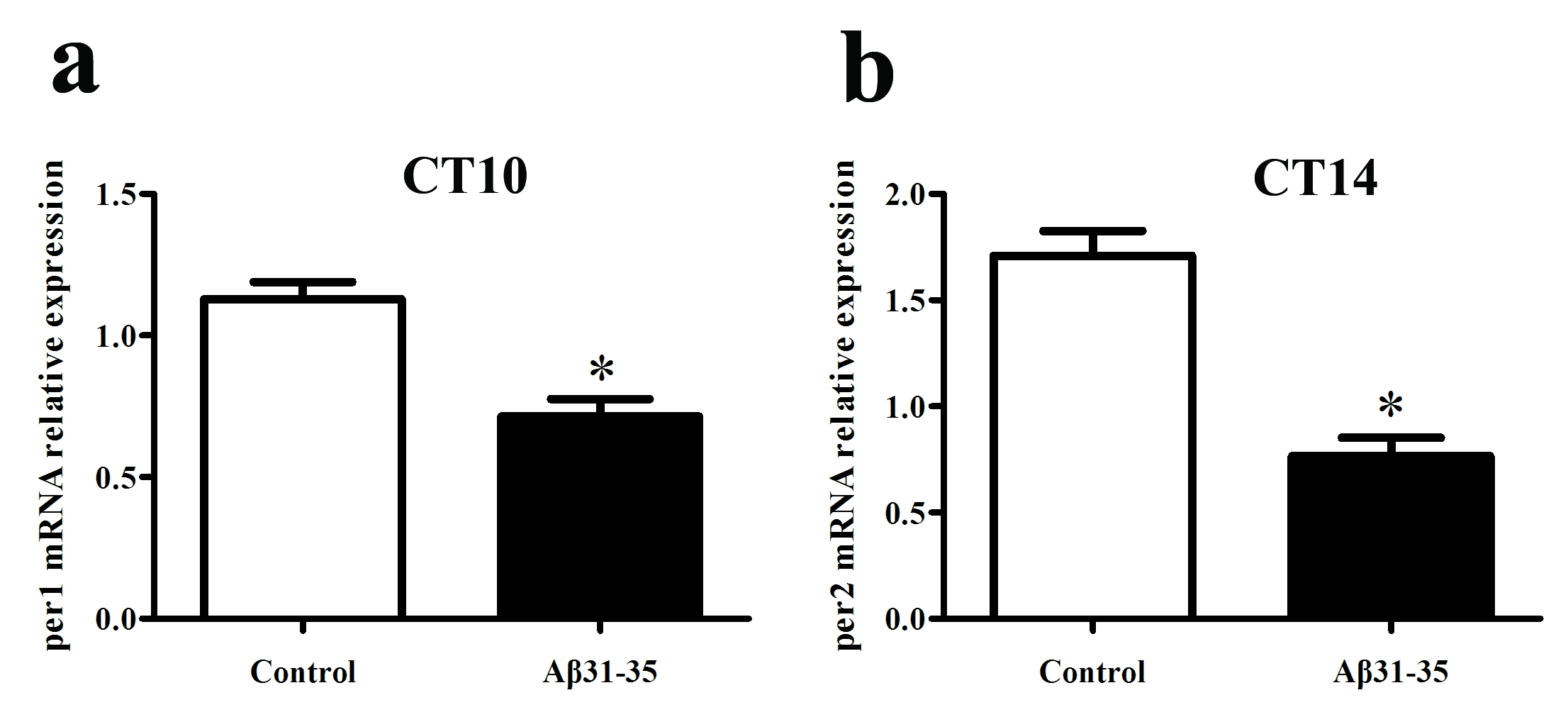

Supplement: Supplementary file 2 — Figure S2. (TIF 1272 kb) [file 13041_2019_432_MOESM2_ESM.tif]
